# Genetic Determinants of RNA Editing Levels of ADAR Targets in *Drosophila melanogaster*

**DOI:** 10.1534/g3.115.024471

**Published:** 2015-12-11

**Authors:** Yerbol Z. Kurmangaliyev, Sammi Ali, Sergey V. Nuzhdin

**Affiliations:** * Department of Biological Sciences, University of Southern California, Los Angeles, California 90089; † Institute for Information Transmission Problems, Moscow, Russia 127994; ‡ Department of Computational Biology, Saint Petersburg Polytechnical University, Saint Petersburg, Russia 195251

**Keywords:** *Drosophila*, RNA editing, natural variation, quantitative trait loci

## Abstract

RNA editing usually affects only a fraction of expressed transcripts and there is a vast amount of variation in editing levels of ADAR (adenosine deaminase, RNA-specific) targets. Here we explore natural genetic variation affecting editing levels of particular sites in 81 natural strains of *Drosophila melanogaster*. The analysis of associations between editing levels and single-nucleotide polymorphisms allows us to map putative *cis*-regulatory regions affecting editing of 16 A-to-I editing sites (*cis*-RNA editing quantitative trait loci or *cis*-edQTLs, *P* < 10^−8^). The observed changes in editing levels are validated by independent molecular technique. All identified regulatory variants are located in close proximity of modulated editing sites. Moreover, colocalized editing sites are often regulated by same loci. Similar to expression and splicing QTL studies, the characterization of edQTLs will greatly expand our understanding of *cis*-regulatory evolution of gene expression.

ADAR-mediated adenosine to inosine deamination (A-to-I editing) is the most widespread type of RNA editing in metazoans. Inosines are recognized by cellular machinery as guanosines, and editing may result in alteration of the encoded proteins (Nishikura 2010). The disruption of proper RNA editing may result in deleterious phenotypes including neural dysfunctions (Palladino *et al.* 2000; Li and Church 2013; Slotkin and Nishikura 2013), and cancer (Qi *et al.* 2014). ADAR enzymes are known to target double-stranded regions of RNA molecules, but the principles determining specificity at particular RNA editing sites, and their editing levels, are poorly understood (Nishikura 2010).

Identification of *cis*-regulatory elements that determine the editing levels of individual sites is essential to unraveling the underlying regulatory mechanisms. Analysis of genetic variation is a powerful approach to study the regulatory mechanisms underlying various phenotypic traits. Previously, analysis of population-level transcriptomic data has revealed widespread natural variation in gene expression and splicing profiles. Genome-wide associations between this variation and whole genome sequences can be used to map the putative regulatory variants affecting these traits. This approach was successfully utilized for mapping of expression and splicing quantitative trait loci (Pickrell *et al.* 2010; Montgomery *et al.* 2010; Lappalainen *et al.* 2013; Battle *et al.* 2014; Kurmangaliyev *et al.* 2015). It has been shown previously that the genetic variation in editing enzymes may be correlated with the editing level of target genes (Hassan *et al.* 2014).

Very recently, Ramaswami and colleagues used targeted RNA sequencing to map putative *cis*-regulatory variants associated with changes in editing levels at 600 genomic loci of *Drosophila melanogaster* (*cis*-RNA editing quantitative trait loci or *cis*-edQTL), and found a sizeable fraction of them significant at nominal false discovery rate (FDR) level (Ramaswami *et al.* 2015). Here we analyzed whole-transcriptome data from 81 *D. melanogaster* strains and identified, using much more stringent criteria, only 11 edQTLs associated with editing levels of 16 A-to-I sites (*P* < 10^−8^). While several strong effect edQTLs were replicated in both studies, the concordance was incomplete. Importantly, we used Sanger sequencing experiments to validate our analysis, and to address any concerns regarding the technical biases in the analysis of RNA editing based on short read data. We conclude that the identified edQTLs represent a novel type of functional genetic variation affecting the editing levels of individual target sites of ADAR.

## Materials and Methods

### Genotypes and transcriptomes

The genotyping data for 216 inbred strains of *D. melanogaster* was downloaded from NCBI SRA (PRJNA36679; PRJNA74721). The analyzed strains represent two natural populations [Raleigh, North Carolina (Mackay *et al.* 2012) and Winters, California (Campo *et al.* 2013)], and the w1118 line (tester strain). The single nucleotide polymorphisms (SNPs) were named using GATK (McKenna *et al.* 2010) as described previously (Campo *et al.* 2013). These variants were used for filtering RNA editing sites overlapping with polymorphic positions and for edQTN mapping (described below).

The transcriptomic data for 81 F1-hybrids are available at NCBI SRA (PRJNA281652). These hybrids were generated by crossing 81 inbred strains (the subset of 216 described above) with one common tester line (w1118). Total mRNA from adult female heads was sequenced on the Illumina HiSeq2000 platform (Kurmangaliyev *et al.* 2015). Replicates corresponding to the same F1-hybrids were merged and analyzed together. Full list of strains used in this study is in Supporting Information, Table S1.

Paired-end RNA-Seq reads (2 × 100 bp) were mapped to the reference *D. melanogaste*r genome (dm3/BDGP 5.72) using STAR (Dobin *et al.* 2013). Duplicated reads were removed using Picard MarkDuplicates (http://broadinstitute.github.io/picard/). For further analysis we used only uniquely and concordantly mapped reads.

### RNA editing levels of A-to-I editing sites

The set of 5302 previously identified A-to-I editing sites was compiled from the results of four recent studies (Graveley *et al.* 2011; Rodriguez *et al.* 2012; Ramaswami *et al.* 2013; St Laurent *et al.* 2013). Further, we filtered out editing sites that were overlapping SNPs observed in 216 *Drosophila* strains. Additionally, we removed editing sites that were overlapping with annotated repetitive regions, and sites from intergenic and heterochromatic regions (Kent *et al.* 2002).

We used mapped RNA-Seq reads to quantify each site’s editing level in each F1-hybrid. To this end, we calculated RNA editing levels for each site as the fraction of G-nucleotides among total count of A- and G-nucleotides overlapping a given genomic position. RNA editing levels were estimated only for sites covered by at least 20 reads, otherwise it was considered as nonavailable (NA) for given editing sites in a given sample. Further analysis was performed on editing sites with estimated editing levels in at least 40 F1-hybrids. The filtered set comprised 1619 editing sites in 597 genes. The full list of editing sites is provided in Table S2.

### edQTN mapping

Association tests between RNA editing level and proximal SNPs were performed using EMMAX (Kang *et al.* 2010). The calculation of identity-by-state (IBS) kinship matrix and association tests were performed on SNPs with minor allele frequency (MAF) ≥ 0.05. For each editing site, we tested only proximal SNPs that were located within 100,000 bp upstream or downstream of the analyzed site. RNA editing levels were transformed using a rank-based inverse normal transformation.

**Figure 1:**
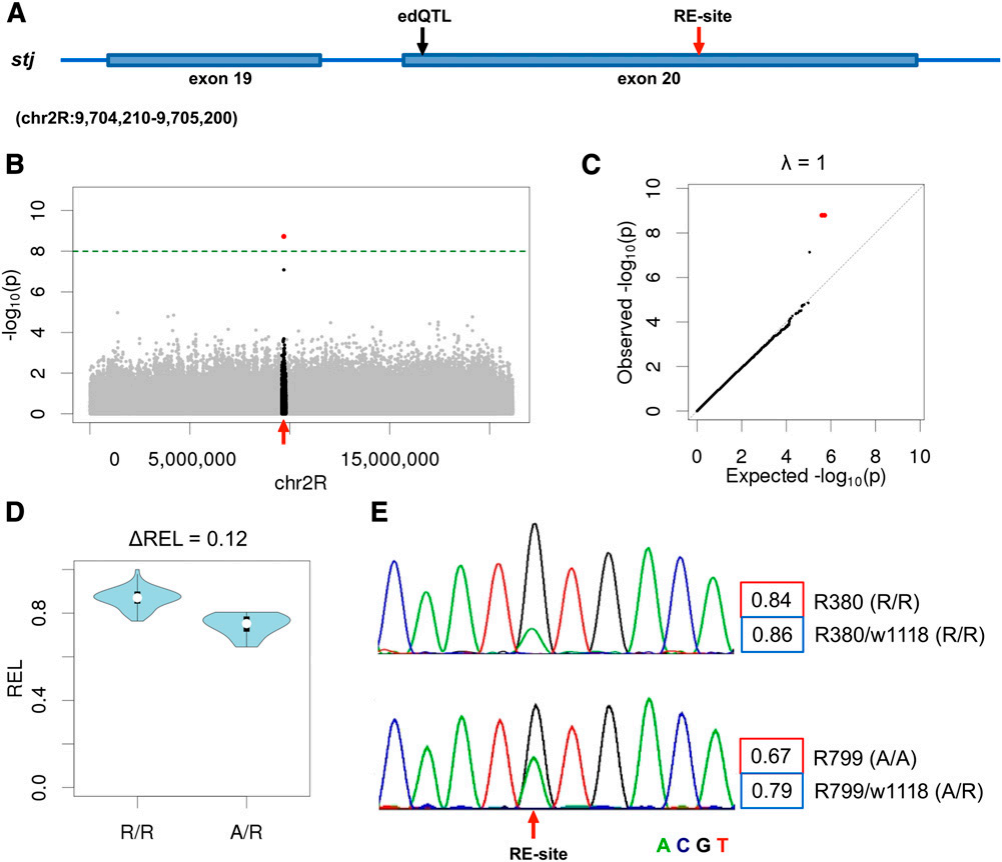
RNA editing quantitative trait nucleotides (edQTN) in gene *stj*. (A) The position of edQTN and associated RNA editing site on *stj* gene. (B) Manhattan plot of association *P*-values for all SNPs from the same chromosome arm. The SNPs in 200-kb region around editing site are highlighted in black. The dashed green horizontal line indicates the Bonferroni corrected significance threshold corresponding to the primary association tests (*P* < 0.05). Significantly associated SNPs are highlighted in red. The position of the RNA editing site is marked by a red arrow. (C) Quantile–quantile plot for observed and expected distributions of association *P*-values (for the whole chromosome). The genome-wide inflation factor (λ) is indicated. (D) Distributions of RNA editing levels in F1-hybrids carrying reference (R/R) and alternative alleles (A/R) of edQTN (all F1-hybrids carried one copy of a reference allele from common tester line (w1118)). (E) Sanger sequencing chromatograms of RNA editing site for two inbred strains that carried only reference (R380 – R/R) or alternative alleles (R799 – A/A) of edQTN. RNA editing levels estimates based on Sanger sequencing and RNA-Seq data are shown in red and blue boxes, respectively (see *Materials and Methods*). Corresponding genotypes of edQTN in sequenced inbred lines and F1-hybrids are also indicated. Similar plots for all identified editing site/edQTN associations are provided in Figure S2, Figure S3, Figure S4, Figure S5, Figure S6, Figure S7, Figure S8, Figure S9, Figure S10, Figure S11, Figure S12, Figure S13, Figure S14, Figure S15, and Figure S16.

To test for genome-wide inflation of false-positive results, we performed additional association tests for each edQTN-associated editing site. To this end, we performed association tests of given editing sites with all SNPs on the same chromosome arms. The resulting *P*-values were used for creation of Manhattan and Q-Q plots, and for calculation of genome-wide inflation factors (λ) (Figure 1, Figure S2, Figure S3, Figure S4, Figure S5, Figure S6, Figure S7, Figure S8, Figure S9, Figure S10, Figure S11, Figure S12, Figure S13, Figure S14, Figure S15, and Figure S16). λ was defined as ratio of medians of observed and expected uniform log *P*-value distributions. Statistically significant associations are provided in Table S3 (Bonferroni-corrected *P* < 0.05).

RNA editing sites that were associated with the same SNP (or block of linked SNPs) were grouped into the clusters (Table 1). For each cluster, we chose one putative causal variant with the lowest association *P*-value (top edQTN). For Cluster 8, only one of the edQTNs was located in the same gene as the regulated site, and this SNP was considered as top edQTN.

**Table 1:** RNA editing sites and associated edQTNs

Cluster	Gene	Chr	RE-Site	Region	Top edQTN	*P*-Value	Distance	edQTN-Region	∆REL
1	*IA-2*	chr2L	1010857	UTR	1010859 (1)	8.2×10^−9^	2	Same exon	0.10
2	*sky*	chr2L	20872840	UTR	20872520 (1)	3.2×10^−9^	320	Same exon	0.08
3	*prom*	chr2R	20306770	CDS	20307490 (2)	2.2×10^−10^	720	Adjacent intron	0.16
			20306773			1.6×10^−11^	717		0.14
4	*stj*	chr2R	9704884	CDS	9704591 (2)	1.6×10^−9^	293	Same exon	0.12
5	*Gβ76C*	chr3L	19682867	UTR	19682869 (3)	1.8×10^−12^	2	Same exon	0.07
			19682970			9.0×10^−12^	101		0.44
			19682971			5.7×10^−12^	102		0.40
6	*CG42540*	chr3L	4590708	UTR	4590740 (1)	2.7×10^−9^	32	Same exon	0.13
7	*CG42540*	chr3L	4591222	UTR	4591093 (1)	2.7×10^−9^	129	Same exon	0.05
8	*rtp*	chr3R	1061931	UTR	1061987* (124)	2.7×10^−9^	56	Same exon	0.04
			1062097			1.3×10^−9^	110		0.23
			1062100			4.5×10^−11^	113		0.19
8	*unc79*	chr3R	15064567	CDS	15064546 (1)	1.6×10^−10^	21	Same exon	0.14
10	*Cpn*	chr3R	7990069	CDS	7990072 (5)	3.2×10^−9^	3	Same exon	0.08
11	*Sh*	chrX	17832044	intron	17832426 (1)	9.8×10^−14^	382	Same intron	0.06

Cluster, the group of colocalized editing sites jointly associated with the same edQTNs; RE-Site, the position of editing sites; Region, the genic location of RNA editing sites; Top edQTN, the SNP with the most significant association (the numbers in parentheses represent total number of SNPs associated with editing sites in a given cluster); *P*-value, *P*-value of association with top edQTN is indicated; distance, the distance between editing sites and top edQTN in bp; edQTN-region, the location of edQTN relative to the editing sites. ∆REL: effect size. * – in the Cluster 8, the top edQTN is the SNP that was located within the same gene with editing sites (see main text for details)

### Sanger sequencing

Sanger sequencing was used to validate editing level estimates based on RNA-Seq data. For each edQTN-associated editing site, we designed a pair of primers to cover corresponding regions of the transcript (450–660 bp). Closely-associated editing sites were analyzed using the same amplicons. For each validated edQTN we chose two strains that carried two alternate alleles of a given SNP (Table S4). The validations were performed on homozygous inbred strains.

Total RNA was extracted from 10 adult female heads using a Direct-zol kit (Zymogen). cDNA templates were generated using Superscript III First-Strand Synthesis System (Life Technologies). Afterward, cDNA templates were amplified using the Phusion High-Fidelity PCR Kit (NEB). PCR products were confirmed, by running 2% agarose gels, purified and submitted for Sanger sequencing (Laragen, Culver City, CA). The primers and specific annealing conditions are provided in Table S5. The correlation between editing level estimates based on RNA-Seq data and Sanger sequencing are shown in Figure S1. Sequencing chromatograms were visualized using Chromas Lite (http://technelysium.com.au/). The RNA editing level was calculated as the ratio of the height of the G-peak over the sum of heights of G- and A-peaks. Chromatograms are shown in Figure 1E, Figure S2, Figure S3, Figure S4, Figure S5, Figure S6, Figure S7, Figure S8, Figure S9, Figure S10, Figure S11, Figure S12, Figure S13, Figure S14, Figure S15, and Figure S16.

### Data availability

Genomic and transcriptomic data analyzed in this study is available at NCBI SRA (PRJNA36679; PRJNA74721; PRJNA 281652).

## Results and Discussion

Here we studied the genetic variation of editing levels of A-to-I sites among 81 natural strains of *D. melanogaster*. We analyzed the set of editing sites that were identified in four recent large-scale transcriptomic studies (Graveley *et al.* 2011; Rodriguez *et al.* 2012; Ramaswami *et al.* 2013; St Laurent *et al.* 2013). The editing levels of these sites were measured in transcriptomes of F1 crosses between a variety of wild-type inbred strains and a common tester line (Nuzhdin *et al.* 2012; Kurmangaliyev *et al.* 2015). RNA editing level was estimated based on whole-genome RNA-Seq data as the fraction of G-nucleotides among total count of A- and G-nucleotides overlapping an edited position. After filtering candidate editing sites based on location (known SNPs, repetitive elements, intergenic, heterochromatic), and filtering for coverage the data set contained 1619 editing sites in 597 genes.

These editing level estimates were used as quantitative traits to map putative regulatory variants associated with differences in RNA editing. The analyzed natural strains of *D. melanogaster* are from two natural populations [Raleigh, North Carolina (Mackay *et al.* 2012) and Winters, California (Campo *et al.* 2013)]. To account for potentially confounding population structure, association tests were run using EMMAX (Kang *et al.* 2010). It has been shown that the editing levels are mainly determined by *cis*-genetic effects (Sapiro *et al.* 2015). Therefore we focused only on proximal SNPs located within a 100-kb region upstream or downstream from the analyzed editing site. Overall, we performed association tests between 4,783,794 RNA editing site/SNP pairs. We detected 285 significant associations (Bonferroni-corrected *P* < 0.05, Table S3). Hereafter, we refer to the variants associated with changes in RNA editing levels as RNA editing quantitative trait nucleotides (edQTN).

The edQTNs represent associations between RNA editing levels of 16 sites, and 142 proximal SNPs. In eight cases, editing sites were associated with two or more SNPs in linkage disequilibrium (LD) blocks. In three cases, clusters of colocalized editing sites were jointly associated with the same edQTNs (one pair and two trios of colocalized sites). In one case, the cluster of three editing sites was associated with a large LD block spanning several hundred kilobases (Table 1, cluster 8). In summary, we identified 11 associations between clusters of editing sites and edQTNs (Table 1).

Compared to only 14% of tested SNPs, in all but one case, the edQTNs were located in the same genes as the regulated sites. The only exception was a cluster of editing sites in *rtp*, which was associated with a large LD block (Table 1, cluster 8). Only one of the SNPs in this block of QTNs was located in the same gene as the regulated site, which made it the best candidate as a causal variant. In other cases with multiple associated QTNs, SNPs with the lowest association *P*-values were considered as functional candidates (top edQTN, Table 1). Remarkably, all such edQTNs were colocalized in the same exon as associated editing sites or in an adjacent intron. This observation supports the notion that RNA editing is regulated at the level of transcripts.

One representative case is an editing site that represents a protein-recoding event in the gene *stj* (Figure 1). Editing level was associated with two closely linked SNPs located in the same exon, about 290 bp apart from the editing site. (Figure 1A). To control for potential genome-wide inflation of significance we tested and plotted *P*-values for all SNPs from the same chromosome arm. The Manhattan plot and quantile-quantile (Q–Q) plot for observed and expected distributions of association *P*-values are shown in Figure 1, B and C. Similar plots for all identified editing site/edQTN associations are provided in Figure S2, Figure S3, Figure S4, Figure S5, Figure S6, Figure S7, Figure S8, Figure S9, Figure S10, Figure S11, Figure S12, Figure S13, Figure S14, Figure S15, and Figure S16.

We used the difference between median editing level in F1-hybrids that carried two alternate alleles of the edQTN as a measure of effect size (∆REL, Figure 1D). ∆REL–values for identified associations varied from 4 to 44% (Table 1). However, the aforementioned transcriptome data are derived from crosses against a common tester, and true effect size may be underestimated.

The analysis of RNA editing using short reads can be easily confounded with various technical artifacts (Bass *et al.* 2012). The major concern with transcriptome QTL mapping studies is the influence of mapping biases associated with genetic variation. SNPs affect number of mismatches between RNA-Seq reads and reference genome, and this can result in false-positive correlations between genetic variants and measurements based on short reads. Here we used Sanger sequencing as independent validation of identified edQTN-associated changes in editing levels. In contrast to RNA-Seq experiments, Sanger sequencing was performed in homozygous strains. An RNA editing site was sequenced in two inbred strains that carried only reference (R/R, R380) or only alternative alleles (A/A, R799) of the edQTN illustrated in Figure 1. This confirmed that transcripts expressed from alleles that carried the alternative variant of the edQTN showed a reduced level of RNA editing. Moreover, the magnitude of change observed in homozygotes (Sanger) was considerably higher than in F1-hybrids (RNA-Seq).

**Figure 2:**
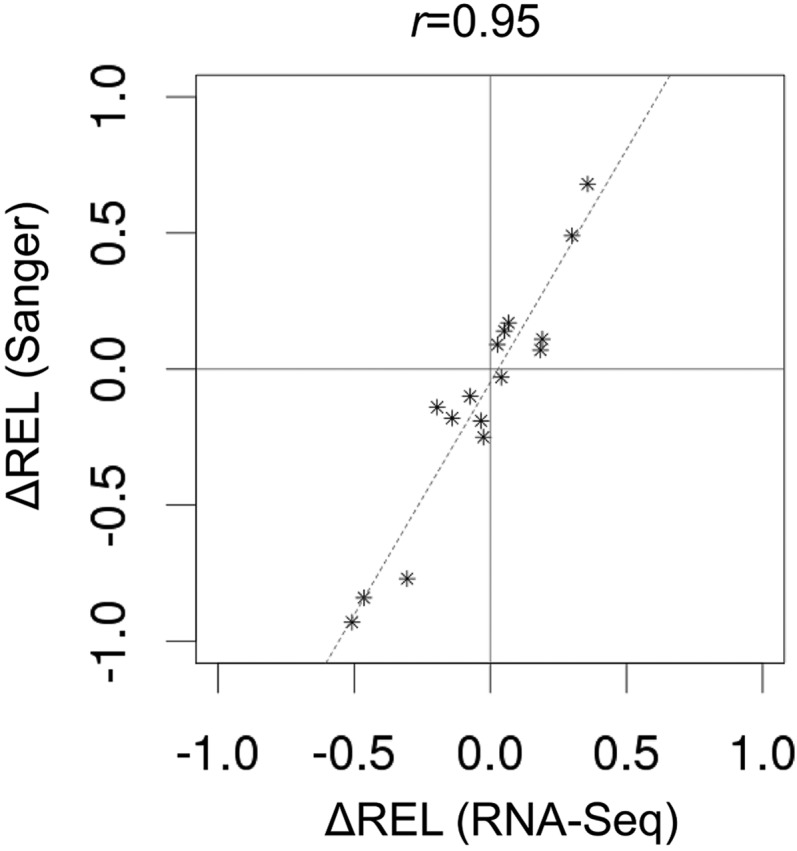
Comparison of edQTN-associated changes in RNA editing estimates based on RNA-Seq data and Sanger sequencing. For each RNA editing site we chose two strains that carried two alternate alleles of edQTN. The direction and magnitude of change was calculated as the difference in editing levels between strains that carried a reference or alternative alleles (∆REL). Note that the RNA-Seq experiments were performed on F1-hybrids, while the Sanger sequencing experiments were performed on homozygous inbred lines (see *Materials and Methods*). The Pearson’s correlation coefficient (*r* = 0.95, *P* < 10^−8^), and line of best fit are indicated (dashed line). Correlation between RNA editing estimates (REL) based on two methods is shown in Figure S1.

Similar validation experiments were performed for all detected editing site/edQTN associations (Figure S2, Figure S3, Figure S4, Figure S5, Figure S6, Figure S7, Figure S8, Figure S9, Figure S10, Figure S11, Figure S12, Figure S13, Figure S14, Figure S15, and Figure S16). Overall, the results of Sanger sequencing were in good agreement with estimates based on RNA-Seq data (Table S4). In 15 of 16 cases the direction of edQTN-associated changes in editing observed by Sanger sequencing agreed with estimates based on RNA-Seq data (Figure 2). Note that the Sanger sequencing experiments were performed on homozygous inbred lines, while the RNA-Seq data were generated from F1-hybrids. Thus, we did not expect an exact match between the RNA editing estimates obtained by each method. Nevertheless, the correlation between editing estimates based on RNA-Seq data and Sanger sequencing was high (Pearson’s *r* = 0.88, *P* < 10^−10^, Figure S1A. In case of F1-hybrids that were homozygous for edQTN (R/R or A/A genotypes in Table S4), the correlation was 0.98 (Pearson’s *r*, *P* < 10^−6^, Figure S1B).

Similar to what we observed for genetic variation in splicing (Kurmangaliyev *et al.* 2013; Kurmangaliyev *et al.* 2015), variation in RNA editing was more prominent in untranslated regions of the genes. Indeed, only 5 of the 16 (31%) edQTN-associated editing sites were located in coding regions (in contrast to 67% among all tested editing sites, Fisher’s exact test, *P* = 0.0001). These sites are codon-recoding events that lead to changes in the protein sequences of four genes. These genes were involved in eye functions [*prom* (Zelhof *et al.* 2006), and *cpn* (Ballinger *et al.* 1993)], neurotransmission [*stj*, (Ly *et al.* 2008)] and behavior [*unc79*, (Lear *et al.* 2013)]. A part of these protein-recoding events had relatively high mean editing levels (∼75%–85%), which is suggestive of functional importance (Pinto *et al.* 2014). This may indicate potential phenotypic consequences for some of the identified edQTN-associated changes in RNA editing.

RNA editing is often mediated through formation of imperfect double-stranded RNA structures between edited regions and complementary sequences in adjacent regions of the transcripts (Nishikura 2010; Reenan 2005). Here, all identified edQTNs were located in close proximity to regulated editing sites, with a median distance of 106\bp. In all cases, edQTNs were located in the same exons or an adjacent intron (Table 1). This implies that identified edQTNs most likely represent mutations in the *cis*-regulatory regions involved in the formation of the local structural determinants required for RNA editing. However, we were unable to detect any direct complementarity around identified pairs of editing sites and edQTN. Nevertheless, mutations at edQTNs may still affect the general structural context of edited regions. This is consistent with the fact that editing at colocalized sites is often associated with same edQTNs.

In a recent study, Ramaswami and colleagues applied a targeted mmPCR-Seq assay to measure RNA editing at 789 A-to-I sites in 131 *D. melanogaster* strains, and were able to identify 353 *cis*-edQTLs at FDR of 5% (Ramaswami *et al.* 2015). Such discrepancy in numbers of edQTLs reported in two studies is primarily a result of difference in used significance thresholds, and strength of reported associations. At FDR of 5% used in Ramaswami *et al.* (2015) the nominal *P*-values of significant associations were up to *P* = 0.004, and the median effect size of reported edQTLs was 2%. In our study, we applied the conservative Bonferroni-corrected significance threshold *P* < 0.05, which corresponded to the nominal association *P*-values of *P* < 10^−8^, and the median effect size of edQTLs was 12%. We compared the lists of putative regulatory variants identified in two studies, and found three strong effect edQTLs detected in both studies. The effect sizes of edQTLs replicated between these two studies were similar (Table S6). In addition, we applied less stringent significance threshold for association *P*-values of *P* < 10^−5^ (which corresponds to genome-wide FDR of 5%), and generated an extended list of significant associations. The extended list of putative edQTNs consists of associations between 78 RNA editing sites and 539 SNPs (Table S7), and 15 of these associations were also significant in Ramaswami *et al.* (2015), (Table S6). This comparison was complicated by the fact that Ramaswami *et al.* (2015) reported only one significantly associated variant per each identified edQTL, while in some cases edQTLs may be represented by multiple SNPs in a large LD blocks. At the same time, these studies were performed on different sets of strains and editing sites, and thus we did not expect replication of all identified strong effect QTL. In general, the associations reported in Ramaswami *et al.* (2015) tended to also have low *P*-values in our analysis (Figure S17). With all these differences, we are pleased to note that the overall results of these two studies obtained using different techniques and approaches complement and confirm each other.

In summary, we were able to map and validate 11 edQTLs associated with changes in editing levels of 16 A-to-I sites (*P* < 10^−8^). We observed that a single locus frequently affects editing of several clustered sites. The low level of LD in fruit flies allowed us to map most of the putative regulatory variants at single nucleotide resolution. This revealed that causal variants affecting editing are most commonly located in the same genic regions as the editing sites. This may be useful to account in future studies of organisms with more complex haplotype structures. Similar to expression and splicing QTL studies, the identification of edQTLs will greatly expand our understanding of gene regulation. The increasing availability of population-level transcriptome data will make similar studies possible in humans and other organisms.

## Supplementary Material

Supporting Information
