# Supplementary material for: Genetic Determinants of RNA Editing Levels of ADAR Targets in Drosophila melanogaster
Source: G3 (Bethesda). 2015 Dec 11;6(2):391–6. doi: 10.1534/g3.115.024471 (PMC4751558; doi:10.1534/g3.115.024471)
Supplement: Supporting Information [file supp_6_2_391__index.html]

Genetic Determinants of RNA Editing Levels of ADAR Targets in Drosophila melanogaster — Genetic Determinants of RNA Editing Levels of ADAR Targets in Drosophila melanogaster — Supporting Information 

# Genetic Determinants of RNA Editing Levels of ADAR Targets in *Drosophila melanogaster*

## Supporting Information for Kurmangaliyev, Ali, and Nuzhdin, 2016

**Files in this Data Supplement:**

- Figure S1 - Correlation between RNA editing level estimates based on sanger sequencing and RNA-Seq data. (.pdf, 98 KB)
- Figure S9 - edQTN in gene *CG42540* (chr3L: 4590708). (.pdf, 108 KB)
- Figure S10 - edQTN in gene *CG42540* (chr3L: 4591222). (.pdf, 109 KB)
- Figure S11 - edQTN in gene *rtp* (chr3R: 1061931). (.pdf, 109 KB)
- Figure S13 - edQTN in gene *rtp* (chr3R: 1062100). (.pdf, 108 KB)
- Figure S14 - edQTN in gene *unc79* (chr3R: 15064567). (.pdf, 114 KB)
- Figure S15 - edQTN in gene *Cpn* (chr3R: 7990069). (.pdf, 110 KB)
- Figure S16 - edQTN in gene *Sh* (chrX: 17832044). (.pdf, 109 KB)
- Figure S17 - Distribution of p-values for editing site/SNP associations that were reported in (Ramaswami et al. 2015) as significant. (.pdf, 53 KB)
- Figure S12 - edQTN in gene *rtp* (chr3R: 1062097). (.pdf, 109 KB)
- Figure S2 - edQTN in gene *IA-2* (chr2L:1010857). (.pdf, 109 KB)
- Figure S3 - edQTN in gene *sky* (chr2L: 20872840). (.pdf, 106 KB)
- Figure S4 - edQTN in gene *prom* (chr2R: 20306770). (.pdf, 108 KB)
- Figure S5 - edQTN in gene *prom* (chr2R: 20306773). (.pdf, 107 KB)
- Figure S6 - edQTN in gene *Gβ76C* (chr3L: 19682867). (.pdf, 111 KB)
- Figure S7 - edQTN in gene *Gβ76C* (chr3L: 19682970). (.pdf, 112 KB)
- Figure S8 - edQTN in gene *Gβ76C* (chr3L: 19682971). (.pdf, 111 KB)
- Table S1 - List of strains used in the study. (.xlsx, 14 KB)
- Table S2 - The set of A-to-I editing sites used in this study. (.xlsx, 345 KB)
- Table S3 - RNA editing sites and associated edQTNs (P<10-8). (.xlsx, 22 KB)
- Table S4 - RNA editing level estimates based on RNA-Seq data and Sanger sequencing. (.xlsx, 11 KB)
- Table S5 - The list of primers and specific annealing conditions use in Sanger sequencing experiments. (.xlsx, 10 KB)
- Table S6 - edQTNs identified both in this study and in (Ramaswami et al. 2015). (.xlsx, 11 KB)
- Table S7 - Extended list of RNA editing sites and associated edQTNs at less stringent significance threshold (P<10-5). (.xlsx, 46 KB)
