# Supplementary material for: Genetic Determinants of RNA Editing Levels of ADAR Targets in Drosophila melanogaster
Source: G3 (Bethesda). 2015 Dec 11;6(2):391–6. doi: 10.1534/g3.115.024471 (PMC4751558; doi:10.1534/g3.115.024471)
Supplement: Supporting Information [file supp_g3.115.024471_FigureS1.pdf]

Supp. Fig. 1

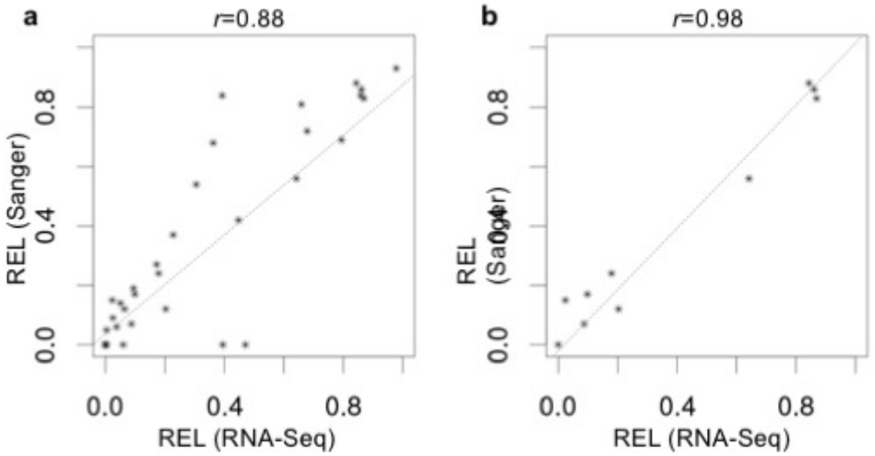

Figure S1. Correlation between RNA editing level estimates based on sanger sequencing and RNA-Seq data. a. For all tested lines. **b.** For F1-hybrids that were homozygous for edQTN (R/R and A/A genotypes in Table S4).
