# Supplementary figures and images for: Genetic Determinants of RNA Editing Levels of ADAR Targets in Drosophila melanogaster
Source: G3 (Bethesda). 2015 Dec 11;6(2):391–6. doi: 10.1534/g3.115.024471 (PMC4751558; doi:10.1534/g3.115.024471)

Supp. Fig. 9: *CG42540* (chr3L: 4590708)

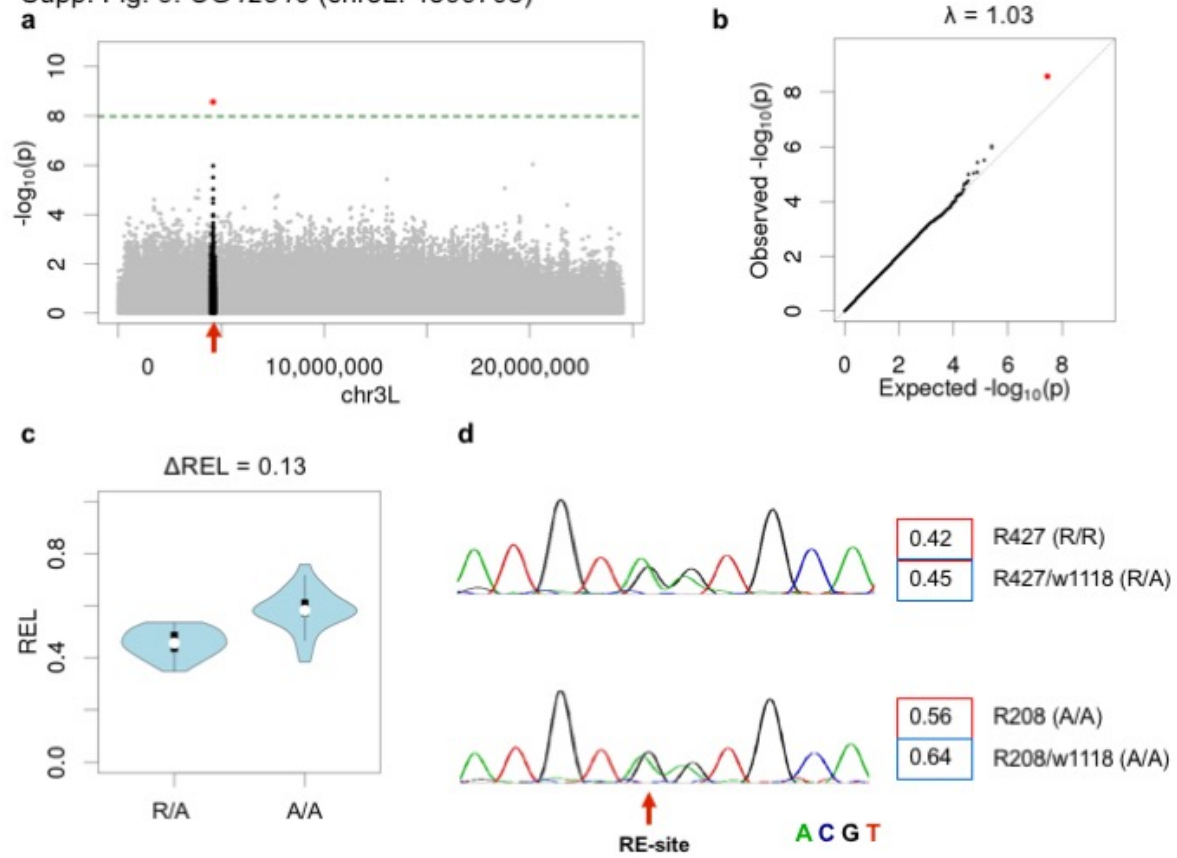

**Figure S9. edQTN in gene *CG42540* (chr3L: 4590708).** See legend of Figure 1 for further details.

Supplement: Supporting Information [file supp_g3.115.024471_FigureS9.pdf]

Supp. Fig. 10: *CG42540* (chr3L: 4591222)

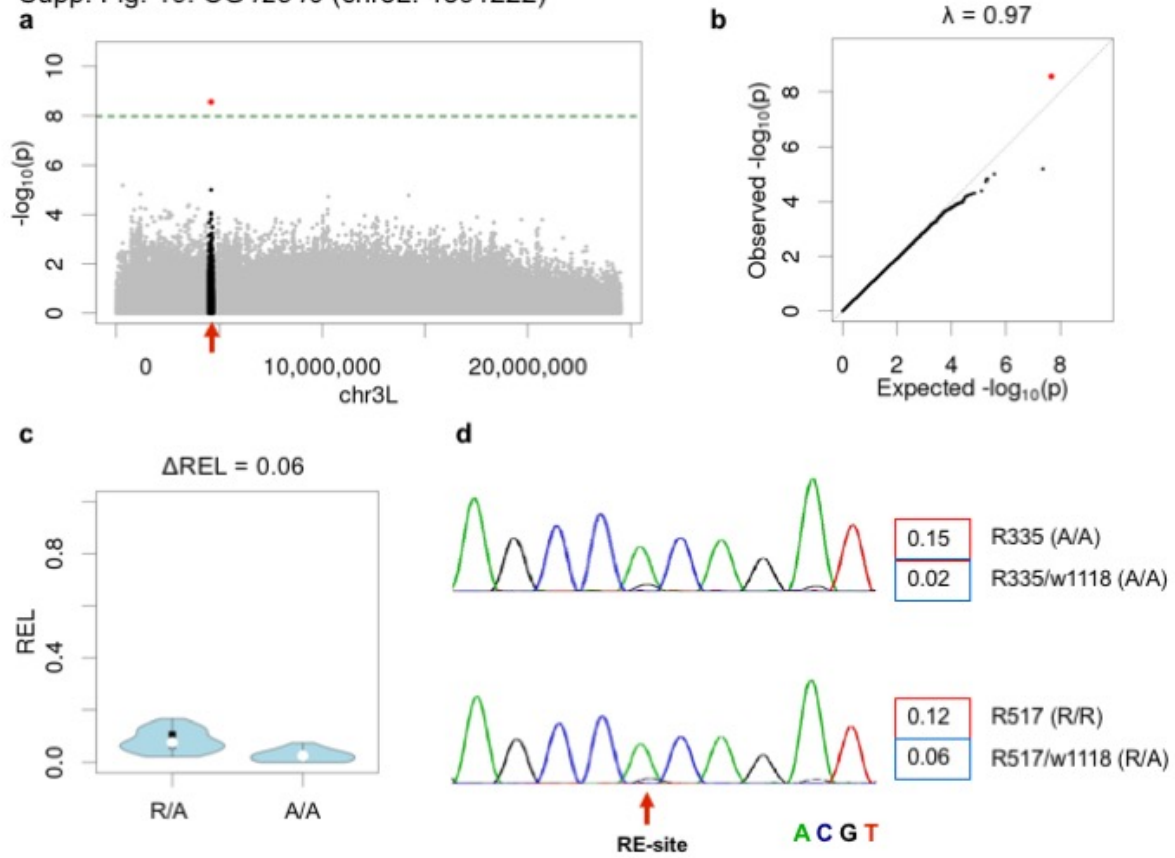

**Figure S10.** edQTN in gene *CG42540* (chr3L: 4591222). See legend of Figure 1 for further details.

Supplement: Supporting Information [file supp_g3.115.024471_FigureS10.pdf]

Supp. Fig. 11: *rtp* (chr3R: 1061931)

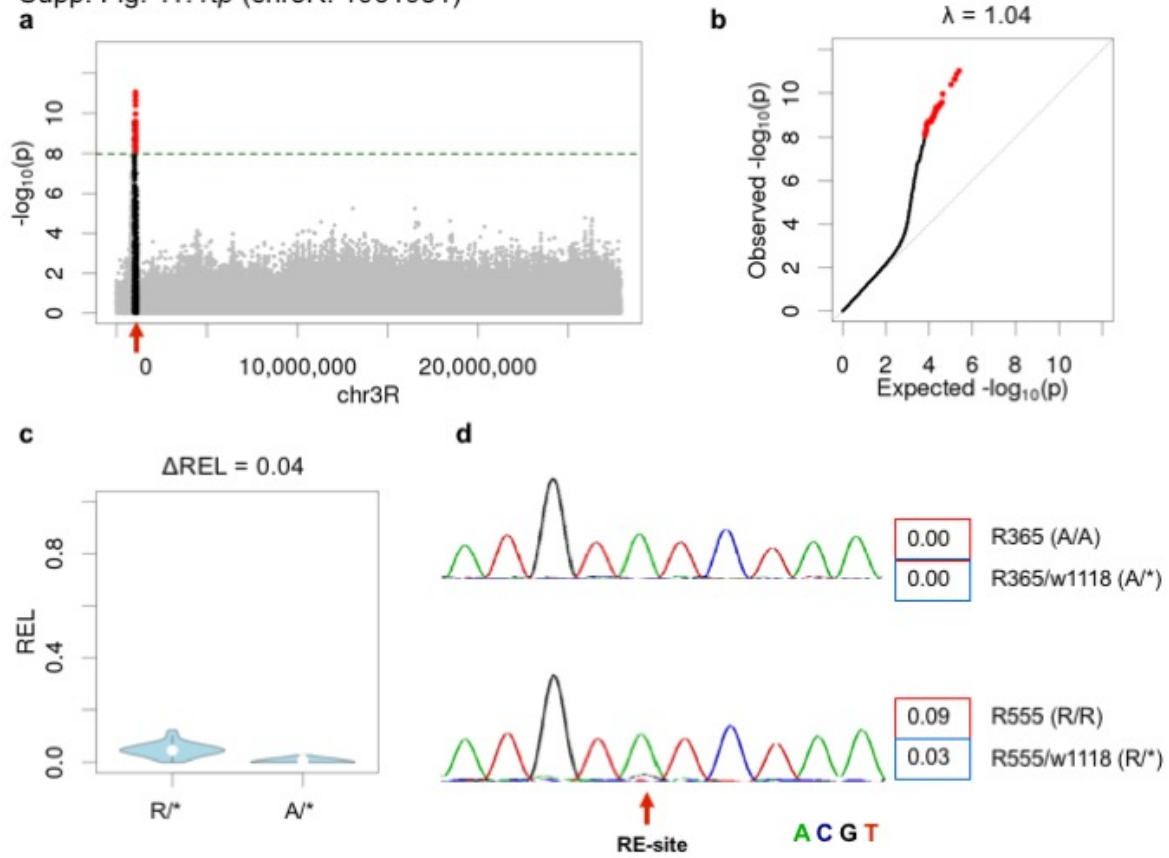

**Figure S11. edQTN in gene *rtp* (chr3R: 1061931).** See legend of Figure 1 for further details.

Supplement: Supporting Information [file supp_g3.115.024471_FigureS11.pdf]

Supp. Fig. 13: *rtp* (chr3R: 1062100)

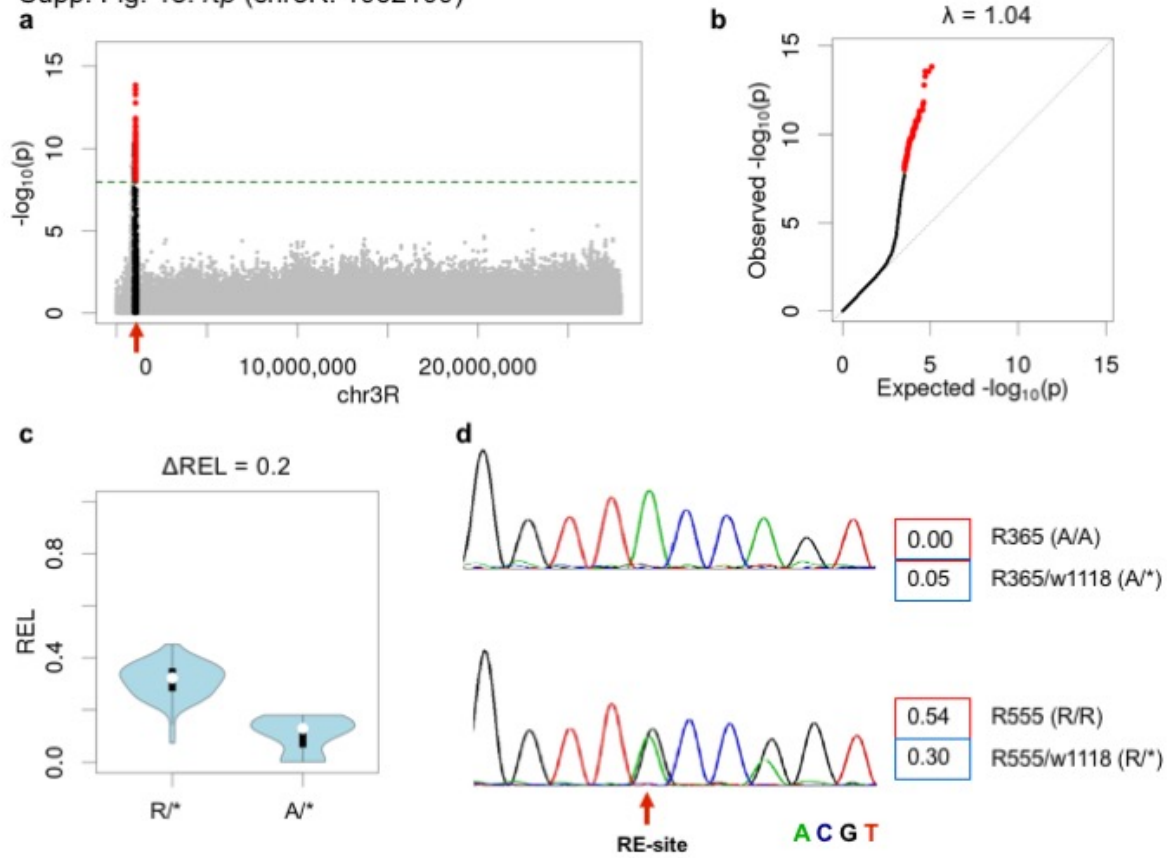

**Figure S13. edQTN in gene *rtp* (chr3R: 1062100).** See legend of Figure 1 for further details.

Supplement: Supporting Information [file supp_g3.115.024471_FigureS13.pdf]

Supp. Fig. 14: *unc79* (chr3R: 15064567)

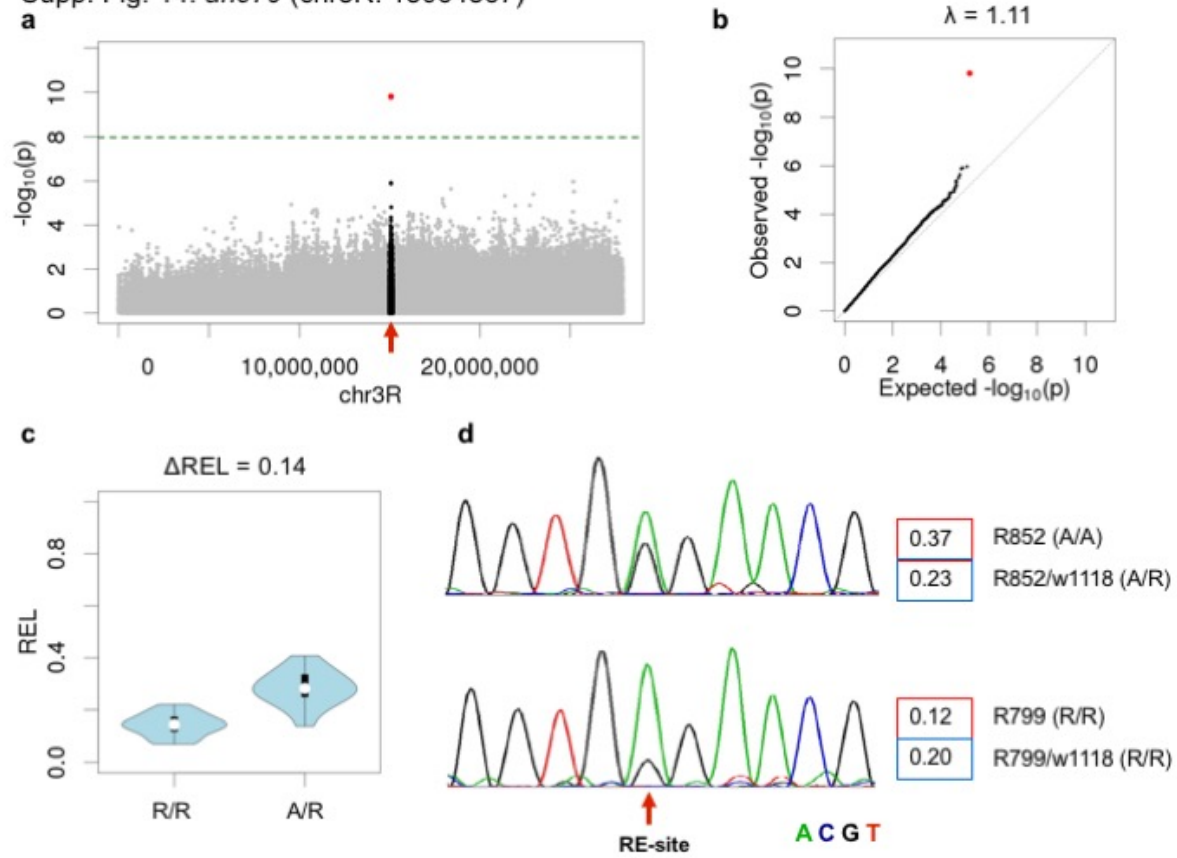

**Figure S14. edQTN in gene *unc79* (chr3R: 15064567).** See legend of Figure 1 for further details.

Supplement: Supporting Information [file supp_g3.115.024471_FigureS14.pdf]

Supp. Fig. 15: *Cpn* (chr3R: 7990069)

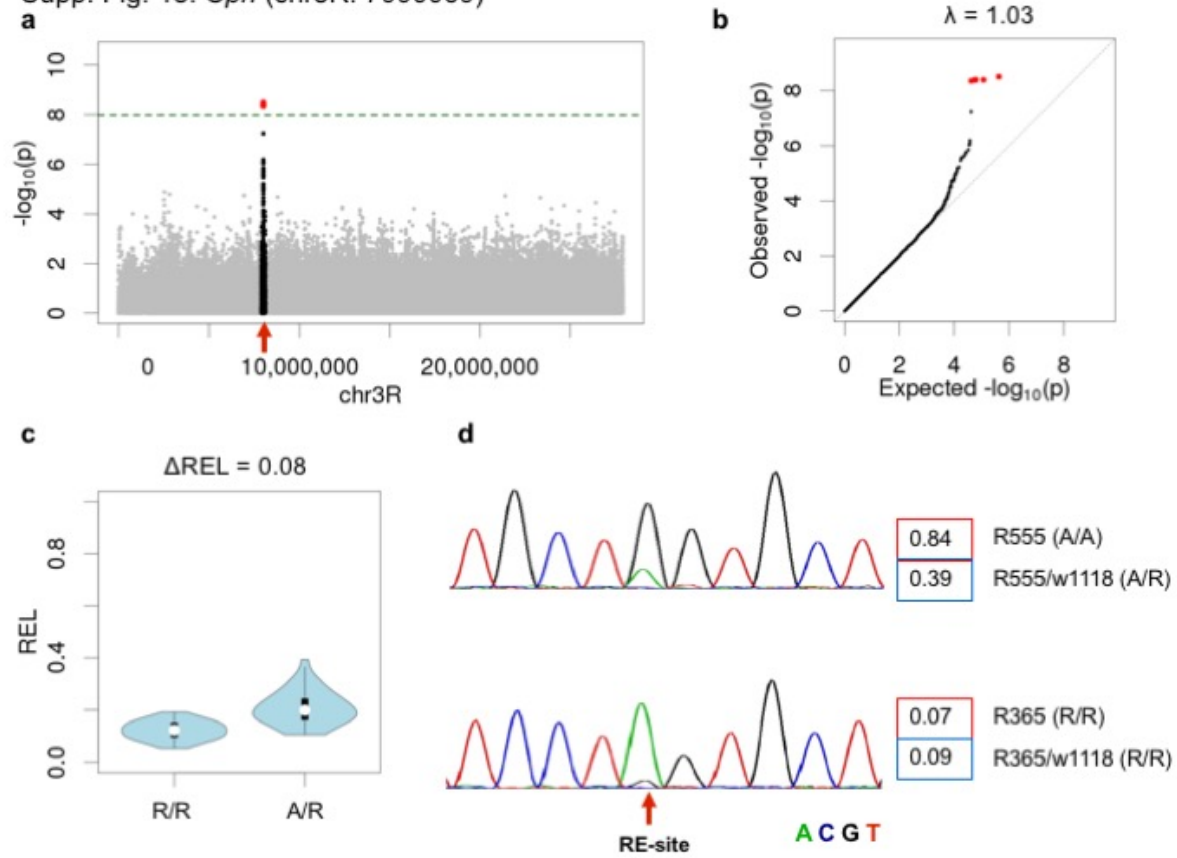

**Figure S15. edQTN in gene *Cpn* (chr3R: 7990069).** See legend of Figure 1 for further details.

Supplement: Supporting Information [file supp_g3.115.024471_FigureS15.pdf]

Supp. Fig. 16: *Sh* (chrX: 17832044)

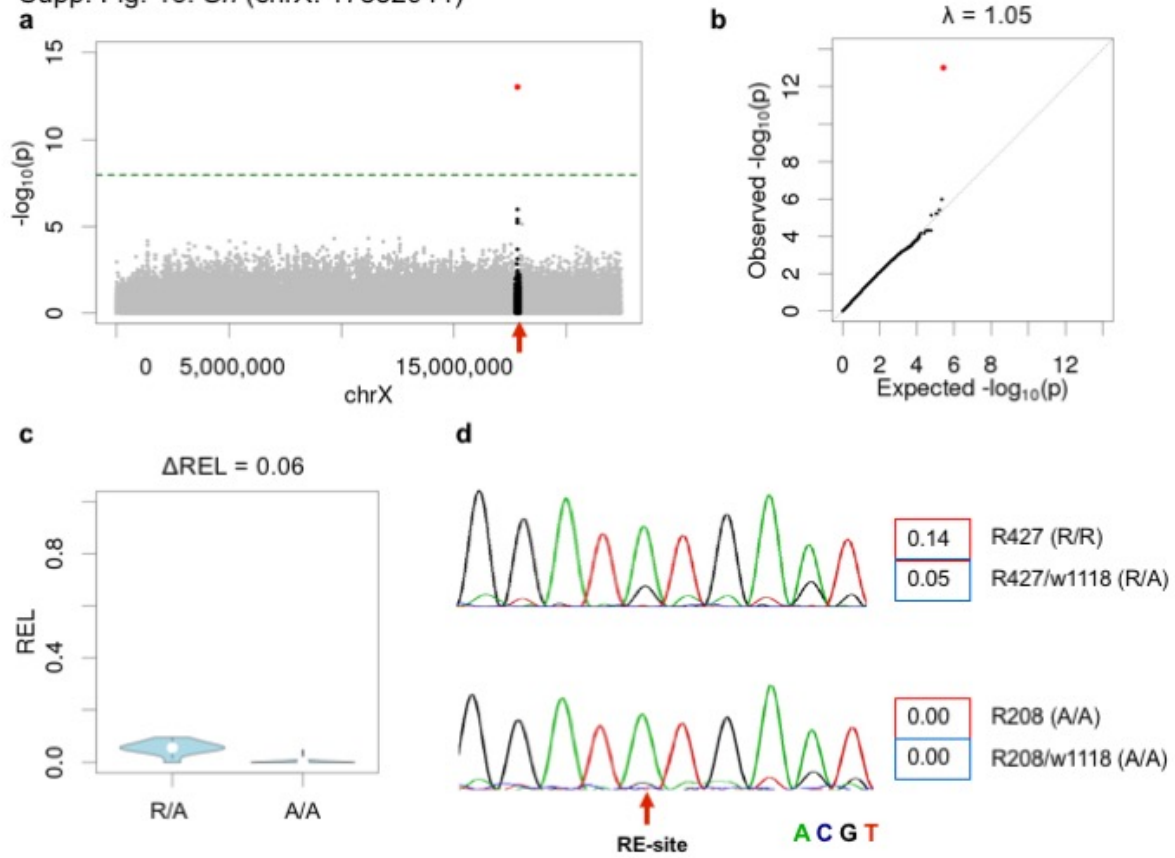

**Figure S16. edQTN in gene *Sh* (chrX: 17832044).** See legend of Figure 1 for further details.

Supplement: Supporting Information [file supp_g3.115.024471_FigureS16.pdf]

Supp. Fig. 12: *rtp* (chr3R: 1062097)

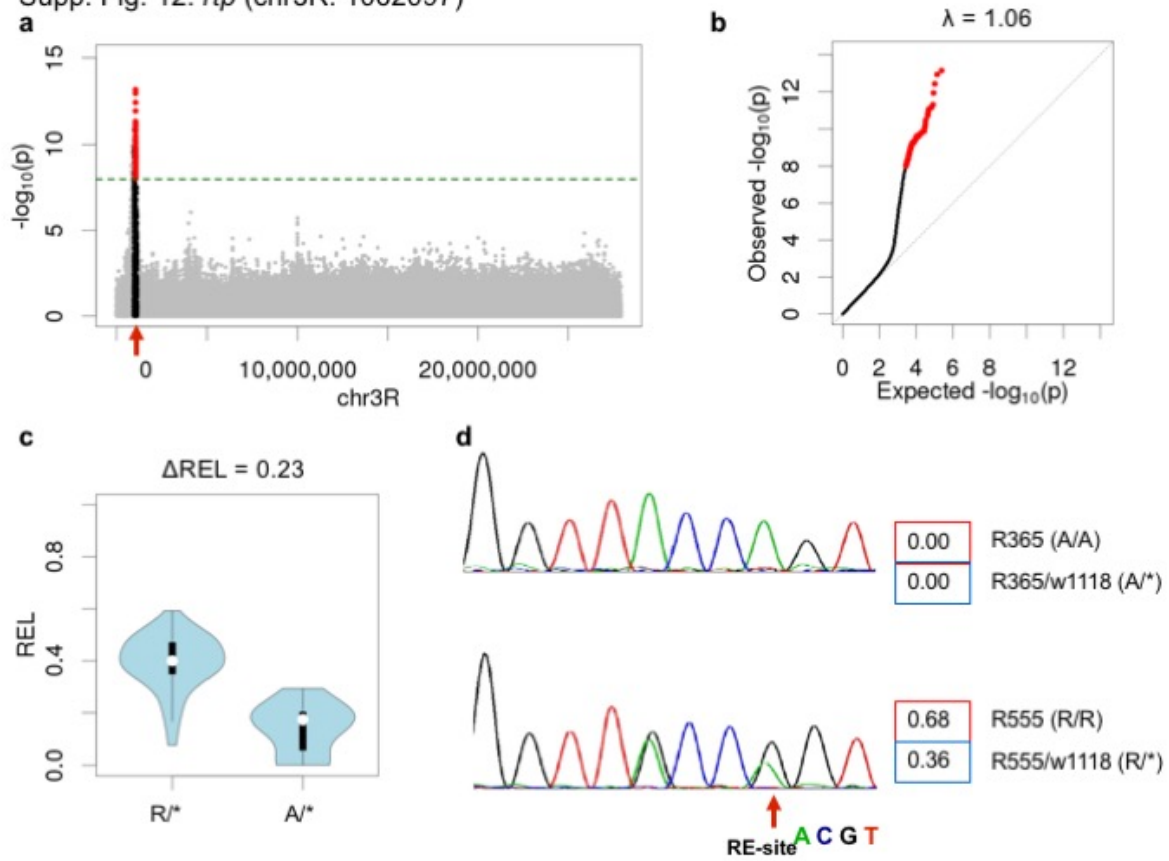

**Figure S12. edQTN in gene *rtp* (chr3R: 1062097).** See legend of Figure 1 for further details.

Supplement: Supporting Information [file supp_g3.115.024471_FigureS12.pdf]

Supp. Fig. 3: *sky* (chr2L: 20872840)

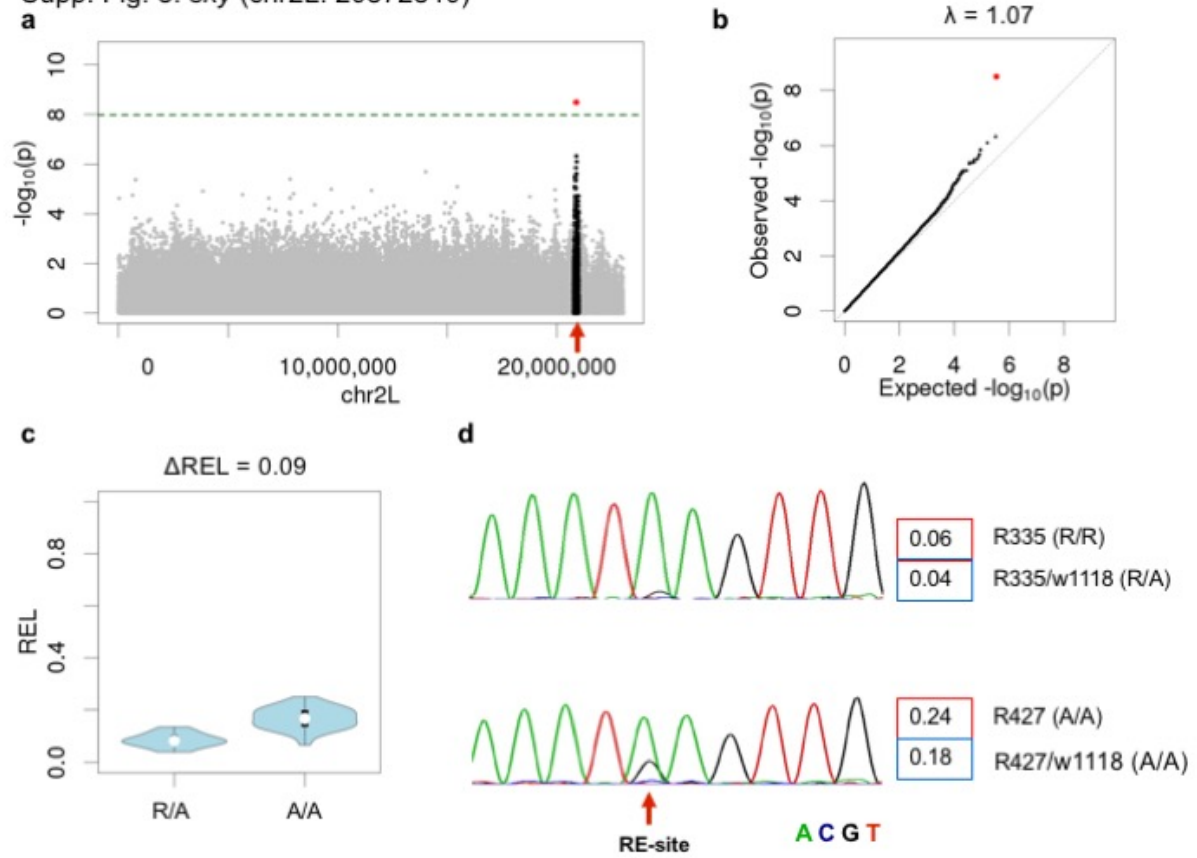

**Figure S3. edQTN in gene *sky* (chr2L: 20872840).** See legend of Figure 1 for further details.

Supplement: Supporting Information [file supp_g3.115.024471_FigureS3.pdf]

Supp. Fig. 4: *prom* (chr2R: 20306770)

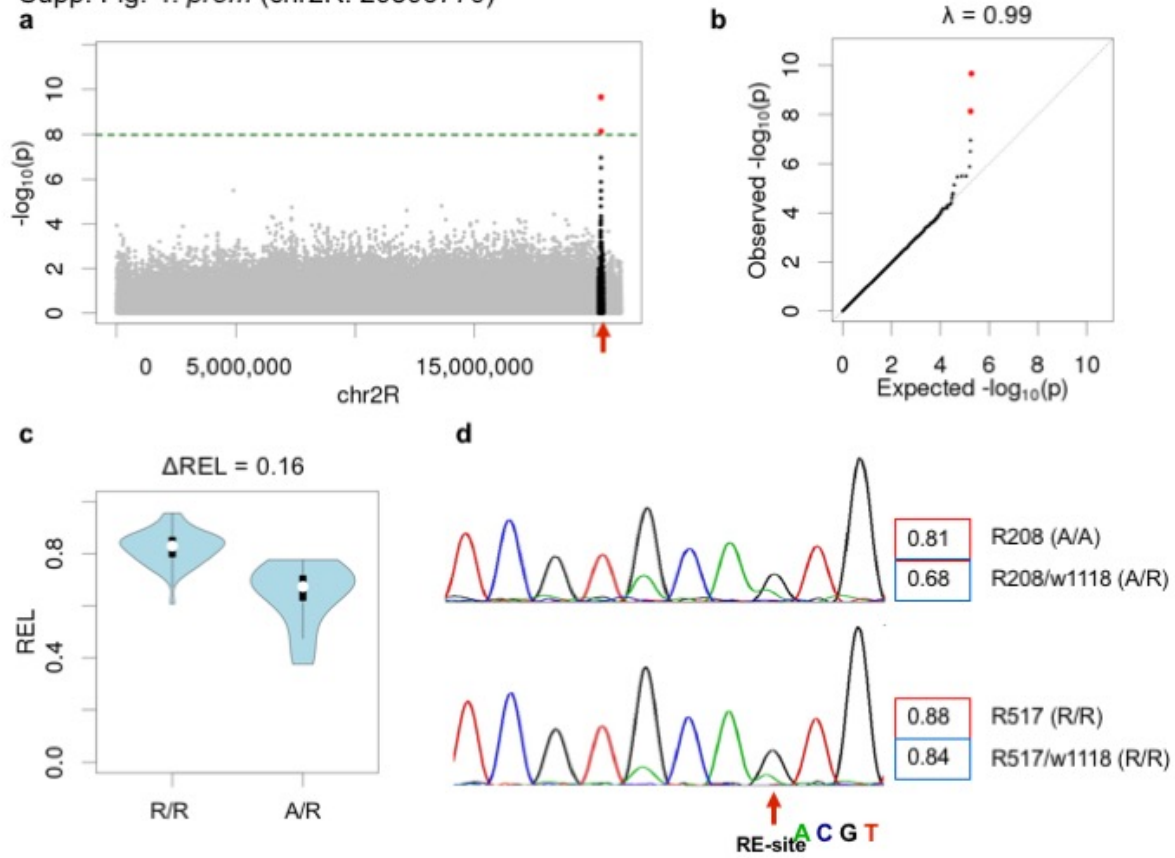

**Figure S4. edQTN in gene *prom* (chr2R: 20306770).** See legend of Figure 1 for further details.

Supplement: Supporting Information [file supp_g3.115.024471_FigureS4.pdf]

Supp. Fig. 5: *prom* (chr2R: 20306773)

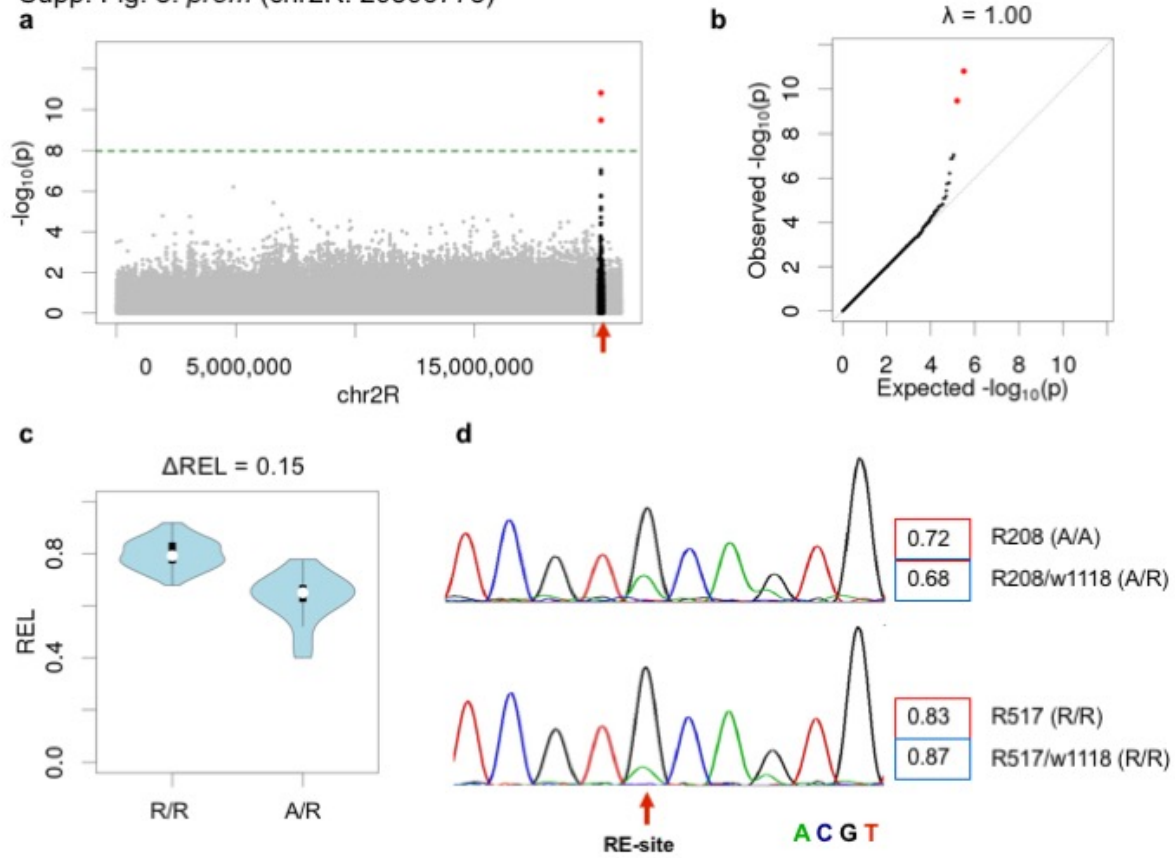

**Figure S5. edQTN in gene *prom* (chr2R: 20306773).** See legend of Figure 1 for further details.

Supplement: Supporting Information [file supp_g3.115.024471_FigureS5.pdf]

Supp. Fig. 6:  $G\beta 76C$  (chr3L: 19682867)

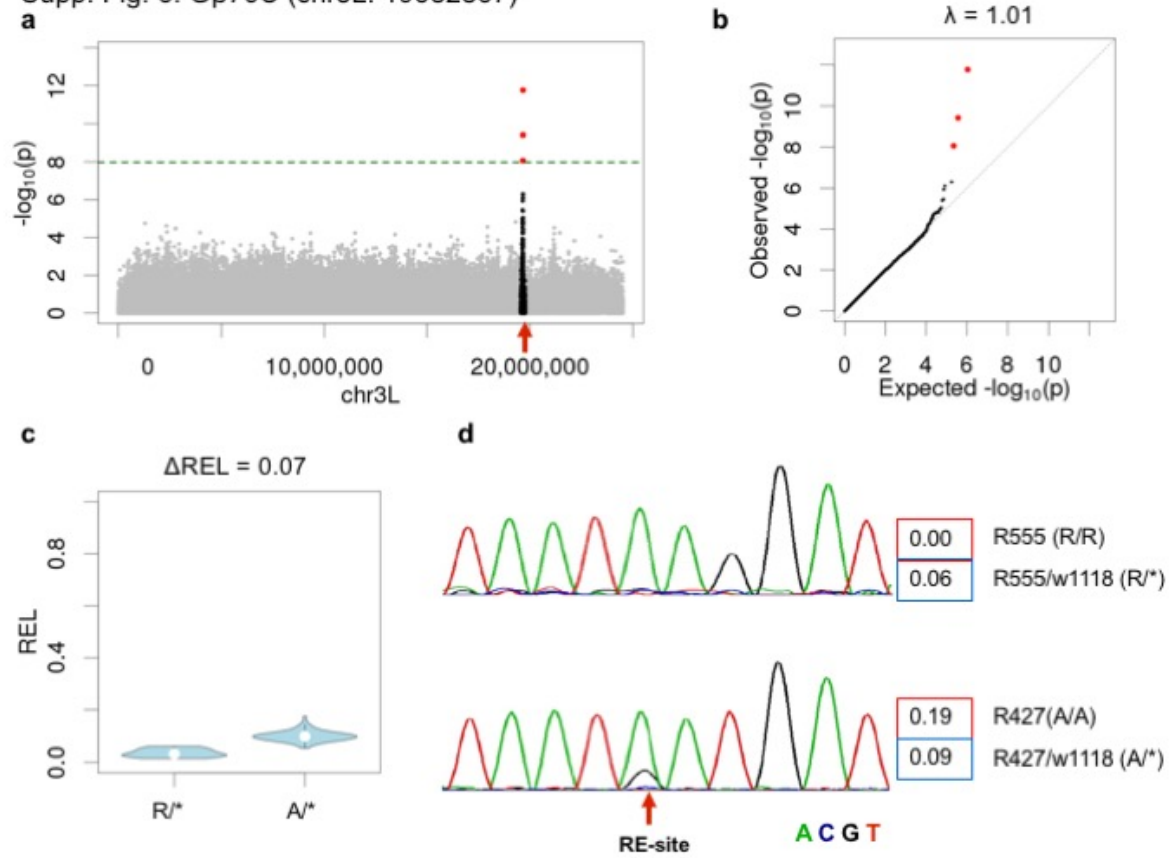

**Figure S6. edQTN in gene  $G\beta 76C$  (chr3L: 19682867).** See legend of Figure 1 for further details.

Supplement: Supporting Information [file supp_g3.115.024471_FigureS6.pdf]

Supp. Fig. 7:  $G\beta 76C$  (chr3L: 19682970)

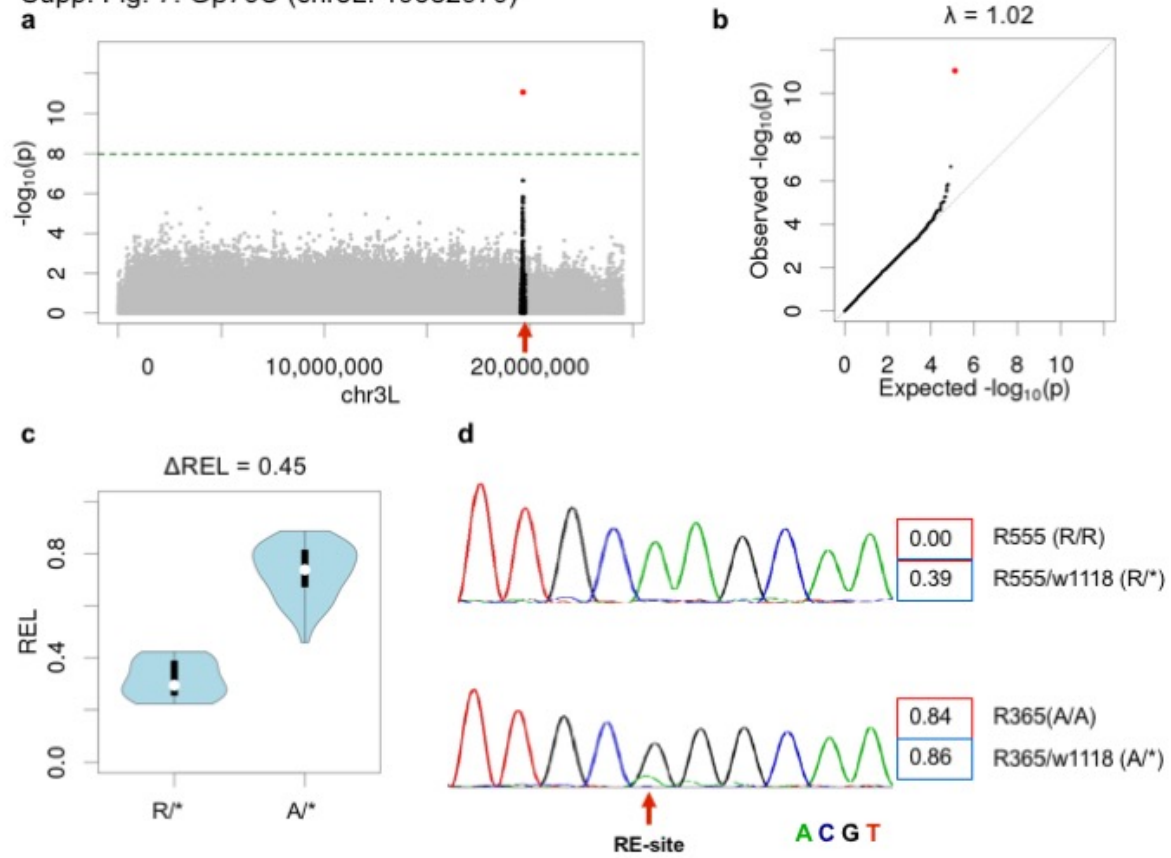

**Figure S7. edQTN in gene  $G\beta 76C$  (chr3L: 19682970).** See legend of Figure 1 for further details.

Supplement: Supporting Information [file supp_g3.115.024471_FigureS7.pdf]

Supp. Fig. 8:  $G\beta 76C$  (chr3L: 19682971)

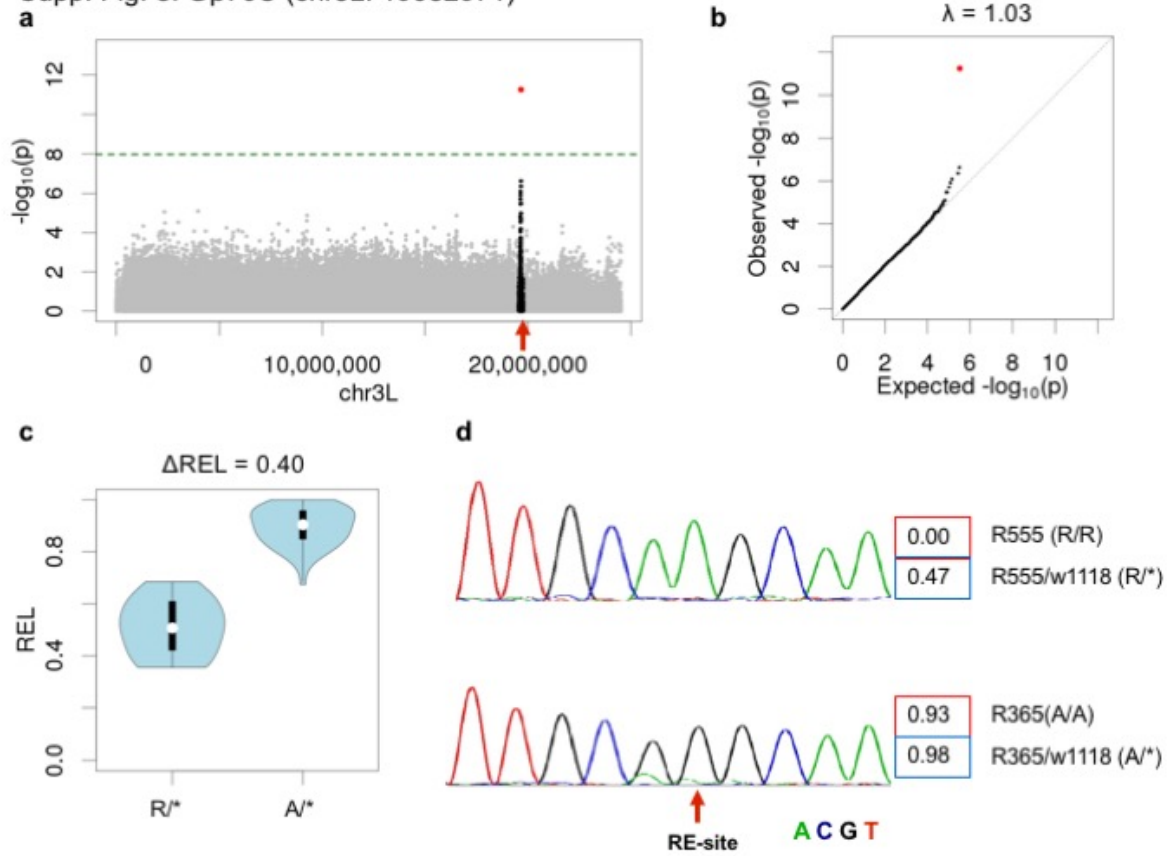

**Figure S8. edQTN in gene  $G\beta 76C$  (chr3L: 19682971).** See legend of Figure 1 for further details.

Supplement: Supporting Information [file supp_g3.115.024471_FigureS8.pdf]
