# Supplementary material for: Genetic Determinants of RNA Editing Levels of ADAR Targets in Drosophila melanogaster
Source: G3 (Bethesda). 2015 Dec 11;6(2):391–6. doi: 10.1534/g3.115.024471 (PMC4751558; doi:10.1534/g3.115.024471)
Supplement: Supporting Information [file supp_g3.115.024471_FigureS17.pdf]

Supp. Fig. 17

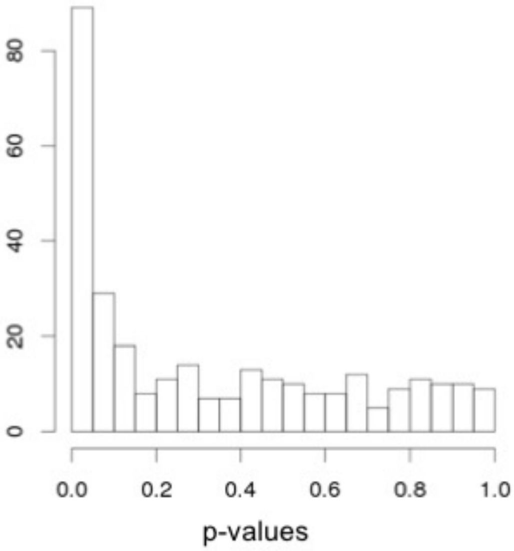

Figure S17. Distribution of p-values for editing site/SNP associations that were reported in (Ramaswami et al. 2015) as significant.
