# Supplementary material for: Genetic Determinants of RNA Editing Levels of ADAR Targets in Drosophila melanogaster
Source: G3 (Bethesda). 2015 Dec 11;6(2):391–6. doi: 10.1534/g3.115.024471 (PMC4751558; doi:10.1534/g3.115.024471)
Supplement: Supporting Information [file supp_g3.115.024471_FigureS2.pdf]

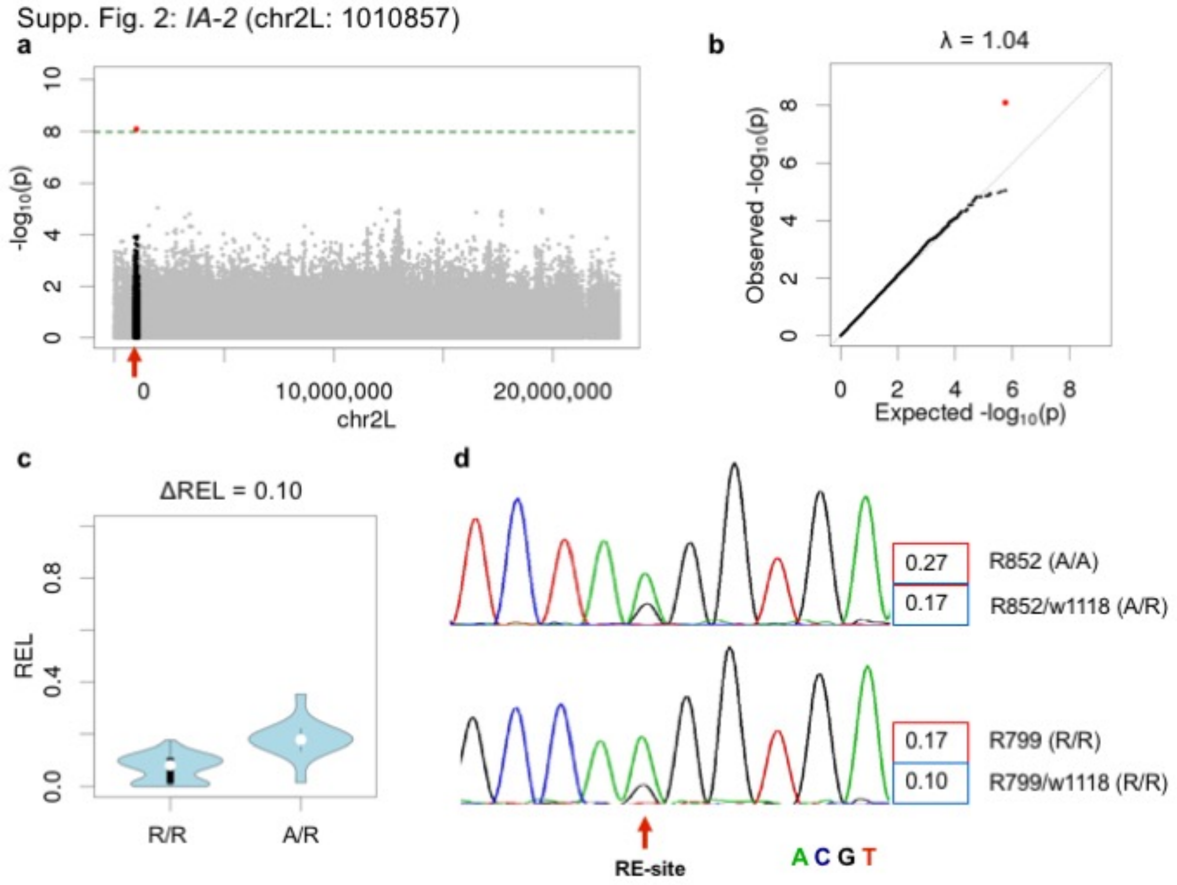

Figure S2. edQTN in gene *IA-2* (chr2L:1010857). The genomic coordinate of editing site is indicated. The Manhattan plot (**a**) and quantile-quantile (**b**) plot for observed and expected distributions of association P-values. **c**. Distributions of RNA editing levels in F1-hybrids carrying two alternate alleles of edQTN. **d.** Sanger sequencing chromatograms of RNA editing site for two inbred strains that carried only reference (R/R) or alternative alleles (A/A). See legend of Figure 1 for further details.
